# The importance of manager support for the mental health and well-being of ambulance personnel

**DOI:** 10.1371/journal.pone.0197802

**Published:** 2018-05-23

**Authors:** Katherine Petrie, Aimée Gayed, Bridget T. Bryan, Mark Deady, Ira Madan, Anita Savic, Zoe Wooldridge, Isabelle Counson, Rafael A. Calvo, Nicholas Glozier, Samuel B. Harvey

**Affiliations:** 1 Black Dog Institute, Sydney, NSW, Australia; 2 School of Psychiatry, University of New South Wales, Sydney, NSW, Australia; 3 Guys and St Thomas NHS Foundation Trust, Occupational Health Department, The Education Centre, St Thomas’ Hospital, London, United Kingdom; 4 King’s College London, London, United Kingdom; 5 Ambulance Victoria, Doncaster, Victoria, Australia; 6 NSW Ambulance, Rozelle, NSW, Australia; 7 School of Electrical and Information Engineering, University of Sydney, Sydney, NSW, Australia; 8 Brain and Mind Centre, University of Sydney, Sydney, NSW, Australia; Örebro University, SWEDEN

## Abstract

Interventions to enhance mental health and well-being within high risk industries such as the emergency services have typically focused on individual-level factors, though there is increasing interest in the role of organisational-level interventions. The aim of this study was to examine the importance of different aspects of manager support in determining the mental health of ambulance personnel. A cross-sectional survey was completed by ambulance personnel across two Australian states (N = 1,622). Demographics, manager support and mental health measures were assessed. Hierarchical multiple linear regressions were conducted to determine the explanatory influence of the employee’s perception of the priority management places upon mental health issues (manager psychosocial safety climate) and managers’ observed behaviours (manager behaviour) on employee common mental disorder and well-being within ambulance personnel. Of the 1,622 participants, 123 (7.6%) were found to be suffering from a likely mental disorder. Manager psychosocial safety climate accounted for a significant amount of the variance in levels of employee common mental health disorder symptoms (13%, p<0.01) and well-being (13%, p<0.01). Manager behaviour had a lesser, but still statistically significant influence upon symptoms of common mental disorder (7% of variance, p<0.01) and well-being (10% of variance, p<0.05). The perceived importance management places on mental health and managers’ actual behaviour are related but distinct concepts, and each appears to impact employee mental health. While the overall variance explained by each factor was limited, the fact that each is potentially modifiable makes this finding important and highlights the significance of organisational and team-level interventions to promote employee well-being within emergency services and other high-risk occupations.

## Introduction

Mental ill health is now the leading cause of long term sickness absence and work incapacity in most high income countries [1–3]. To date, the majority of workplace interventions aiming to enhance employee mental health have largely targeted the individual worker, such as techniques to enhance individual resilience [4–6]. While such individual-level interventions may be important [7], there is growing recognition that workplaces are complex and a number of multi-level models of workplace mental health have been proposed to describe the relationship between factors at an individual, group and organisation-wide level. A recently published framework is an example of one such approach that highlights that there is an increasing need to also consider group and organisational-level factors [8]. Such recommendations are based on the increasing evidence that organisational and psychosocial factors play a critical role in employee mental health and performance [9, 10].

A number of team and organisational-level constructs of workplace support have been proposed. One commonly used construct is “psychosocial safety climate” (PSC). PSC is considered to be an ‘up-stream’ organisational-level resource largely influenced by senior management [11]. PSC refers to an organisation’s policies, practices, and procedures for the protection of employee psychological health and safety, and the level of support and endorsement of these within the organisation. A strong manager PSC is evident when workplace policies are supported and there is a high level of commitment within management to favourable working conditions for employees [12, 13]. A second, related construct is the level of supportive behaviour provided to employees and teams by their supervisor or line manager. This type of direct managerial support has been identified as a key influence upon workplace well-being and performance across a variety of occupations [14]. Whilst these two concepts are conceptually related, they differ in that manager psychosocial safety climate measures the perceived attitudes and commitment to mental health and well-being within the management of the organisation, while the other supportive behaviour considers the actual observed behaviours of managers regarding issues related to mental health. The importance of identifying which, if any, of organisational or leadership variables are associated with adverse mental health outcomes is that many of these are modifiable, meaning they could become the focus of new prevention initiatives.

There are a number of occupations identified as high risk for employee mental ill-health, such as the military and emergency services, mainly due to repeated exposure to potentially traumatic events [15]. Such occupational groups provide an ideal environment in which to examine the potential impact of organisational and leadership factors on mental health outcomes.

Team-level factors have been recognised to play a significant role in employee mental health within the armed forces, clearly an at-risk profession. Studies of UK and US military personnel, have demonstrated that factors including unit cohesion, effective team leadership and unit support are strongly associated with mental health [16–18]. Lower levels of support have been associated with elevated rates of PTSD and depressive symptoms [19, 20], while high levels of support can mitigate risk for poor mental health amongst those exposed to trauma [21, 22]. Higher rates of other common mental disorders (CMD) such as depression and anxiety, and lower levels of well-being are also important outcomes amongst trauma-exposed workgroups [23] and emergency service workers. Ambulance personnel operate within small, team-based contexts in high-stress environments that involve close co-operation between on-duty personnel at local stations. As such, it is likely that similar team and organisational-level variables may also be influential contributors to well-being and performance amongst ambulance personnel.

There is some evidence that ambulance service work may affect mental health negatively in a similar way to that seen amongst military personnel [23, 24]. Ambulance personnel (including paramedics, emergency medical technicians and other ambulance service clinical staff), routinely provide life-saving medical assistance and transport during emergencies. Elevated rates of mental health problems amongst emergency services personnel, particularly post-traumatic stress disorder (PTSD) [25] have been identified. A recent meta-analysis indicated that ambulance personnel may be at particularly high risk compared to other emergency service workers, with ambulance personnel reporting the highest prevalence estimate for PTSD amongst all occupational groups examined at 14.6% [26]. amongst all services. However, research with emergency services is limited, often combines several services into one group, and the prevalence estimates for common mental disorders reported in the literature vary widely [26]. Additionally, ambulance personnel are under-researched and studies of mental health outcomes other than PTSD are rare.

Given evidence that team-level factors such as manager support are important in employee mental health of at-risk groups such as the military, more research is needed in ambulance personnel to examine mental health and workplace risk factors in this at-risk occupation. This study aimed to examine the impact of both manager PSC and manager behaviour on the mental health outcomes amongst a cohort of ambulance personnel. Specifically, we aimed to quantify and describe the relationship between each workplace factor and employee mental health and well-being.

## Method

### Sample

Participants were drawn from two Australian state-wide ambulance services, located in New South Wales and Victoria. Ethical approval was obtained from the South Eastern Sydney Local Health District Human Research Ethics Committee HREC ref no: 16/348 (HREC/16/POWH/684). Within each state, the sample of interest comprised currently employed ambulance personnel providing front-line emergency medical response and out-of-hospital care, patient retrieval and transport. Participants were based at stations across metropolitan and regional/rural areas with an approximately equal distribution sampled across both areas.

### Procedure

All eligible participants were contacted by email with a link to the participant information and consent form, which once completed, directed participants to the online questionnaire. A full copy of the online questionnaire is provided as a supplementary file (S1 File). A reminder email was sent at 7-days to participants who had not yet completed the questionnaire.

### Measures

Demographic characteristics were assessed by asking each participant about their age, gender, location of employment, duration of employment (≤6 months to ≥15 years) and type of employment (full-time, part-time, other categories).

### Manager support variables

#### Manager psychological safety climate

The manager-focused subscales (Management Support and Commitment & Management Priority) of the 12-item Psychosocial Safety Climate Scale (PSC-12) (12) were used to measure employee perceptions of the importance and priority management places upon mental health issues. Both subscales and the overall PSC-12 have demonstrated good reliability, internal consistency, and acceptable validity in a range of occupations (12). An example item is: “Management considers employee psychological health to be as important as productivity.” Responses were provided on a 5-point Likert scale ranging from 1 (“strongly disagree”) to 5 (“strongly agree”), with all responses summed to a total score (range: 6–30). Higher scores indicate a higher degree of perceived commitment to psychosocial safety in an employee’s workplace and greater managerial support for employee mental health. In the current study, internal reliability of these six items was excellent (Cronbach’s alpha of 0.95).

#### Manager behaviour

A total of nine items were developed specifically for this study to assess employee’s direct experience of different aspects of their manager’s behaviour. Items captured employee reports of actual managerial behaviour in the workplace and were worded as follows: “My supervisor… “

1. “… pays attention to my feelings and problems, and notices if I’m not feeling well.”
2. “… shows that they appreciate the way I do my job.”
3. “… helps me with a certain task if necessary.”
4. “… gives me advice on how to handle things if necessary.”
5. “… would be someone I would speak to if I was experiencing workplace stress.”
6. “… is considerate when managing team members.”
7. “… involves me in decision-making.”
8. “… is accessible and approachable to people in the team.”
9. “… remains objective when an issue between staff members arise.”

Collectively these items assess a construct we termed ‘manager behaviour’ (MB). Items were scored on a 5-point Likert scale of frequency from 1 (“never”) to 5 (“always”) and summed to yield a total score (range: 5–25). A less supportive, critical and distant manager (indicated by low scores on these items) can act as a stressor for employees, whilst higher scores reflect more frequent supportive behaviours from managers such as provision of encouragement and resources. In the current study, internal reliability of these nine items was excellent (Cronbach’s alpha of 0.95).

### Mental health variables

#### Symptoms of common mental disorder

Symptoms of CMD were assessed using the 6-item Kessler Psychological Distress Scale or ‘K6’ [27]. The K6 was developed to identify persons with a high likelihood of having a diagnosable mental illness and associated functional limitations. The K6 items assess the frequency of symptoms of general psychological distress, depression and anxiety over the past month. Responses feature a 5-point Likert scale, ranging from “none of the time” (coded 0) to “all of the time” (4). An unweighted summative scoring approach was used in this study that resulted in a total K6 score with a range of 0–24. The K6 scale has demonstrated excellent internal consistency and reliability (Cronbach's alpha = 0.89) [27]. It outperforms many other self-report measures of common mental health symptoms and as a result, has been used in a number of large-scale population health surveys [28–30]. A cut-off score of 13 has previously been used to identify individuals who are likely to be experiencing psychological symptoms of a common mental disorder with significant functional impairment [31]. A K6 score of ≥13 was used as an indicator of “likely CMD” in this study.

*Mental Well-being*: Employee mental well-being was assessed using the Short Warwick-Edinburgh Mental Well-being Scale (SWEMWBS) [32]. This 7-item instrument is well validated in community and clinical samples [32]. The positively-phrased items use a 5-point Likert scale [“none of the time” (1), “rarely” (2), “some of the time” (3), “often” (4), “all of the time” (5)] to assess well-being over the last 2 weeks. Items are summed to provide a total score (range: 7–35), with higher total scores reflecting greater overall psychological well-being for that employee.

### Statistical analysis

All statistical analyses were performed using IBM SPSS statistical software (v22). Descriptive statistics, Chi-square tests, 2-sample independent t-tests and one way ANOVAS were used to examine how representative the current sample was compared to previously published population-based samples. The association between manager psychosocial safety climate (MPSC), manager behaviour (MB), and each of the mental health outcomes was examined using linear regression. Scores for MPSC and MB were each divided into five evenly sized groups (quintiles). Multiple hierarchical linear regression analyses were conducted to assess whether either workplace variable (MPSC and MB) were significant independent predictors of employee mental health outcomes (symptoms of CMD and mental well-being) after adjusting for relevant sociodemographic variables (age range, gender, location, type of employment).

## Results

A total of 1,807 completed online surveys were received. Following removal of blank or duplicate responses (n = 188), eligible survey responses from both locations were combined [NSW (n = 861); Victoria (n = 761)], resulting in a final sample of 1,622 ambulance personnel. The nature of staffing (i.e. rotating rosters, shift-work) made it difficult to determine exact participation rates, although annual staffing figures suggest approximately a quarter of each state-wide service participated in the study (NSW: 27.1%; Victoria: 23.9%).

Table 1 presents a summary of the characteristics for the overall sample. Approximately equal sized groups were obtained from each location (NSW: 53.1%; Victoria: 46.9%) and for each gender (male: 52.2%; female: 47.8%). Chi square tests indicated no significant difference in gender distribution compared to a combined estimate using published figures of both the NSW and Victorian paramedic workforce (p = 0.48). As demonstrated in Table 1, the overall age distribution was similar to that found amongst paramedics in the 2011 Australian census [33]. A total of 123 (7.6%) participants reported mental health symptoms likely to represent the presence of a CMD (K6 score ≥ 13).

**Table 1:** Demographic characteristics and summary scores for both the current sample (N = 1,608) and published population-based samples.

	Current sample	Australian ambulance personnel
**Sociodemographic characteristic**	n (%)	
Age		National data on paramedics^a^
≤20–30 years	300 (18.5%)	20.3%
31–40 years	362 (22.4%)	28.7%
41–50 years	447 (27.6%)	29.4%
51 - ≥60 years	376 (23.2%)	21.5%
Gender ^b^		Local staff records^g^
Male	847 (52.2%)	4,010 (59%)^h^
Female	611 (37.7%)	2,787 (41%)
Location		
Ambulance Victoria (AV)	761 (46.9%)	
NSW Ambulance (NSWA)	861 (53.1%)	
Type of employment		
Full time	1196 (73.7%)	
Part time	154 (9.5%)	
Casual / ACO^c^	69 (4.3%)	
Volunteer / CERT^d^	20 (1.2%)	
On leave (maternity/medical)	11 (0.7%)	
Other^e^ (incl. FWA^f^, contract)	7 (0.4%)	
**Outcome measure**	Average total score (M, SD)	
*Well-being (SWEMWBS; 7-items)*		
Current study	25.25 (4.53)	
*Psychological distress (K6; 6-items)*		
Mean score	5.54 (4.27)	
Likely CMD (K6≥13)^i^		
No	91.6% (1485)	
Yes	7.6% (123)	

^a^ 2011 Australian population census data (unadjusted) on individuals stating ‘paramedic’ as their occupation; comparable age range boundaries used (10–29 years; 30–39 years; 40–49 years, 50–79 years)

^b^ “Prefer not to answer” (n = 28) excluded

^c^ ACO: Ambulance Community Officers (ACOs) are first responders employed on a casual basis.

^d^ CERT: Community Emergency Response Team are volunteers (CERT) who provide basic emergency care services within their local community until the ambulance arrives.

^e^ Other: includes FWA, contract, on-call centre, semi-retired.

^f^ FWA: Flexible Working Arrangement.

^g^ Combined total number of paramedics employed across both locations (Ambulance Victoria staffing figures: 2015–6, for FTE On road Clinical Staff e.g. Paramedics, Team Managers, Patient Transport Officers, Retrieval Registrars, Clinic Transport Officers and Clinical Instructors; NSW Ambulance staffing figures: June 2014, for Ambulance clinical services (mobile) e.g. On-road paramedics).

^h^ Calculated using average % gender distribution across both locations (Ambulance Victoria figures: 2017; NSW Ambulance figures: June 2014)

^i^ Likely common mental disorder (CMD) with significant functional impairment, as indicated by a K6 score of 13 or more.

**Fig 1:**
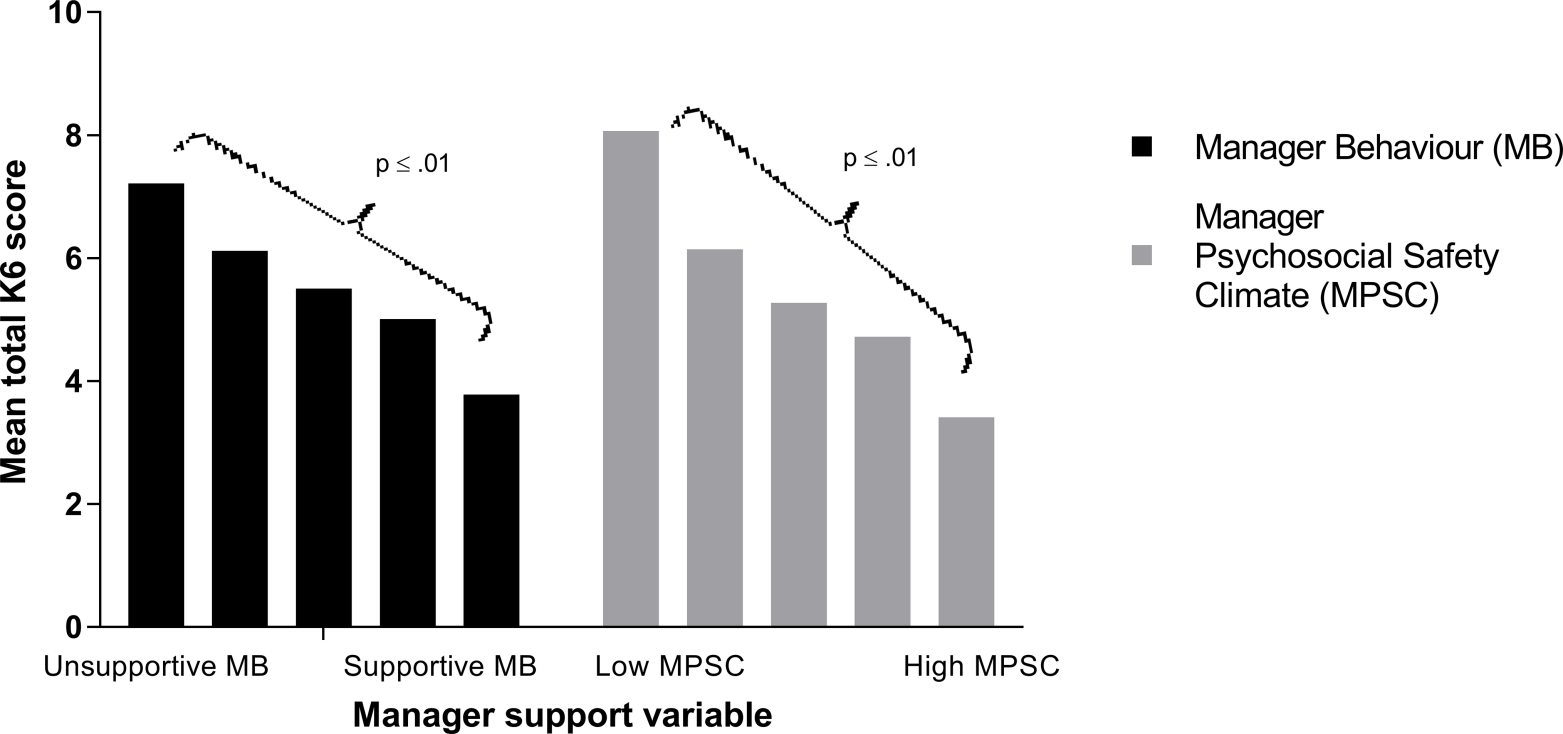
Association between reported level of manager support (manager behaviour and manager psychosocial safety climate) and symptoms of common mental disorder (measured by the total Kessler K6 score). Both MB and MPSC scores have been grouped into quintiles.

**Fig 2:**
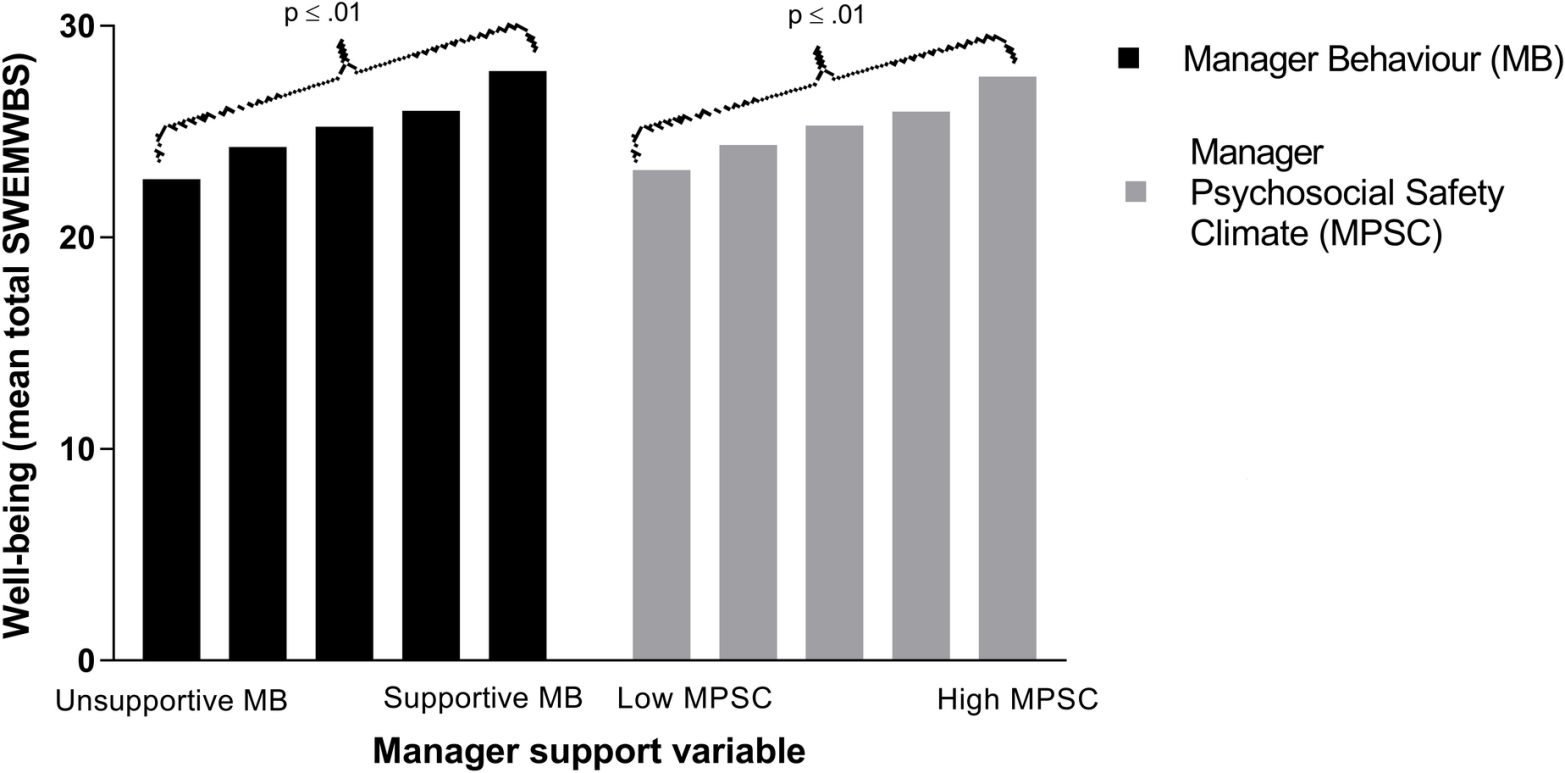
Association between reported level of manager support (manager behaviour and manager psychosocial safety climate) and mental well-being amongst ambulance personnel (Short Warwick-Edinburgh Mental Well-being Scale; SWEMWBS). Both MB and MPSC scores have been grouped into quintiles.

Figs 1 and 2 demonstrate the association between measures of manager support and each of the mental health outcomes. Univariate linear regression confirmed a significant negative linear relationship between both manager psychosocial safety climate and manager behaviour with symptoms of common mental disorder (Fig 1, p<0.01 for both analyses). Similarly, there was a significant positive linear relationship between the two measures of manager support and mental well-being (Fig 2, p<0.01 for both analyses).

Table 2 outlines a series of a priori planned hierarchical regression models investigating the unique influence of manager psychosocial safety climate (MPSC) and manager behaviour (MB) upon each of the mental health outcomes. Initially either MPSC or MB was entered in Model 1 to isolate the role of each of these manager support variables in accounting for the two mental health outcomes of interest. Model 2 adjusted for gender, age range, location, and type of employment. In terms of symptoms of CMD, after adjusting for demographic factors, measures of MPSC accounted for 13% of the variance in employees’ total K6 score (p<0.01). By comparison, measures of MB explained a smaller, though still statistically significant amount of the variance in symptom levels of CMD (sr^2^ = 7%, p<0.01). A similar approach was used to assess the contribution of MPSC and MB to levels of well-being reported by ambulance personnel. These models are also shown in Table 2. After adjusting for demographics, levels of MPSC and MB were able to explain 13% and 10% of the variance in employee mental well-being respectively (p<0.01 for both). All regression models were run a second time post hoc using a selected sample of full-time employees only (n = 1174 valid cases), results were similar with no substantial differences observed (see S1 Table).

**Table 2:** Hierarchical regression analyses modelling the association between manager psychosocial safety climate, manager behaviour and mental health outcomes amongst ambulance personnel.

	Manager Psychosocial Safety Climate (MPSC)			Manager Behaviour (MB)		
	Beta (β)	r^2 /^ sr^2^	p value	Beta (β)	r^2 /^ sr^2^	p value
**Symptoms of CMD (K6)**						
Model 1: Unadjusted	-.368	r^2^ = 0.14	p < .01	Unadjusted -.279	r^2^ = 0.08	p < .01
Model 2: +Dem	-.401	sr^2^ = 0.13	p < .01	+Dem -.272	sr^2^ = 0.07	p < .01
**Well-being (SWEMWBS)**						
Model 1: Unadjusted	.380	r^2^ = 0.14	p < .01	Unadjusted .336	r^2^ = 0.11	p < .01
Model 2: +Dem	.408	sr^2^ = 0.13	p < .01	+Dem .327	sr^2^ = 0.10	p < .05

**Model 1:** Unadjusted.

**Model 2:** Adjusted for demographics (gender, age range, location, type of employment).

## Discussion

This study aimed to examine the influence of two forms of manager support, manager psychosocial safety climate and manager behaviour, upon the mental health of ambulance personnel. As expected from previous studies [24], our results indicated that this sample of ambulance personnel were experiencing measurable symptom burden due to mental ill-health, with 7.6% reporting symptoms indicative of likely common mental disorder. Importantly, in terms of possible interventions for this high-risk group, our study found that both forms of manager support variables were independent and significant predictors of mental health. MB accounted for 10% variance in well-being and 7% variance in symptoms of CMD, whilst MPSC accounted for a larger proportion (13%) of both employee mental health outcomes. These estimates are in line with previous studies that have demonstrated similar-sized contributions of these variables to mental health outcomes within the general workforce [11, 13, 34].

Our findings confirm the importance of manager support as a potentially modifiable factor influencing employee mental health, particularly the related concepts of MPSC and reported MB. The influence of manager support on employee psychological outcomes has been demonstrated in previous research with similar emergency services cohorts [35, 36] and other occupations, though to the best of our knowledge this is the first time the influence of these constructs on mental health has been assessed amongst a sample of ambulance personnel. A somewhat surprising result was the observation that perceptions of managerial PSC and the perceived commitment and culture of the organisation regarding mental health issues, appears to have a more substantial influence on the variance in ambulance personnel well-being and symptoms of CMD compared to the influence of actual observed behaviours of managers. We propose three possible reasons why manager PSC may have a greater impact on employee mental health than manager behaviour. Firstly, PSC is thought to act as a precedent and predictor of a number of well-established workplace risk factors such as bullying and low job control [11, 12]. This breadth of causal influence on multiple variables may give manager PSC an additive or more comprehensive effect than simple behavioural measures. Dollard and colleagues (2010) propose PSC to be the pre-eminent psychosocial risk factor at work due to this influence on other psychosocial risk factors. Following from this, managements’ level of commitment to workers psychological health can also shape actual working conditions and is evident in its operational definition, in the policies, procedures and practices that are implemented and enacted on the ground by management [37]. Secondly, whilst behaviours are invested within a single individual, manager PSC is vested in the entire tier of management and comprises an established set of principles and practices. Whilst direct manager behaviour has a greater degree of variability over time and context, PSC may have a level of sustainability and reach that allows it to have a greater impact at the employee level. Finally, broader psychosocial safety climate constructs have been criticised for their lack of specificity in predicting outcomes [38] with Schneider (2000) proposing that climate measures should be specific to the predicted outcome. Thus, the specific focus of PSC on psychological health may enhance its predictive influence.

When considering the amount of variance in ambulance employee mental health accounted for by the two variables, ranging from 7% to 13%, it is important to consider what this means in terms of their impact as a modifiable risk factor in the workplace. In other words, are the effect sizes we observed clinically important in addition to being statistically significant? We know that common mental disorders such as depression are multifactorial, with multiple non-modifiable risk factors. The three largest contributors to risk for depression identified by large longitudinal twin studies, are: 1) genetic and environmental factors; 2) personality; 3) stressful life events. Each of these factors is estimated to account for a large proportion of the variance in risk for depression; for example, around 29% (men) and 42% (women) [39] and 37% for both genders is related to genetic liability [40] and 63% from individual environmental factors [40], neuroticism is a strong predictor [41] and stressful life events identified as a substantial causal and non-causal predictor [42, 43] of episodes of major depression. Combining and then removing these factors’ high contribution from an individuals’ risk profile leaves only a small amount of risk remaining. Any easily modifiable factors then become incredibly important as targets for interventions to enhance mental health. As such, while MPSC and MB may only be able to explain 7 to 13% of the variance in mental health outcomes amongst ambulance personnel, we suggest that as modifiable factors, they are a crucial target for research and interventions.

Interventions aimed at managers are increasingly considered to be a key component of efforts to improve employee mental health [8]. Manager-based approaches are particularly warranted within at-risk occupations like emergency services and military, which feature strong team-based operations, high-stress roles and elevated rates of mental ill-health. Our results suggest that such interventions need to target both forms of manager support, with the aim of increasing the managerial PSC and improving manager behaviour regarding mental health. However, to date, attempts to evaluate the impact of manager training on manager support have tended to focus on examining self-reported manager behaviour [44–47]. Given the results presented in this study, there is a need to consider how manager training and organisational interventions more generally may need to be tailored to impact manager psychosocial safety climate as well as manager behaviour. Approaches could include developing appropriate policies and procedures and enhancing senior managements’ commitment to and communication regarding employee psychological safety and well-being.

The low response rate is a main weakness of the current study, albeit typical of studies of other similar occupational groups. This creates an issue of potential responder bias, which is particularly problematic for determining an accurate prevalence estimate of CMD in this group. However, given this type of prevalence estimate was never the main aim the study and no comparisons are made with other groups, the impact of this limitation is limited. An additional limitation is the current study’s cross-sectional design that prevents clear interpretation of temporal sequence and duration of these relationships, particularly given the relapsing/remitting course often followed by mental disorders. While the available prospective studies support the temporal precedence of adverse psychosocial work exposures prior to the onset of psychological problems [48], the possibility of reverse causation remains—via alterations in the evaluation of the work environment by those with CMD or through real changes in the workplace environment in response to mental health symptoms [49]. Measurement limitations include self-report assessment, particularly the use of a subjective measure to assess symptoms of CMD that cannot provide the diagnostic precision of a clinical interview. The potential for residual confounding from unmeasured factors, such as personality, may have led to an overestimation of the importance of manager support variables upon mental health outcomes. Selection bias, or the ‘healthy worker effect’, may have influenced the results and their generalisability, given that the emergency services undertake pre-employment screening and rigorous physical training. Finally, these results are drawn from a specific population and profession and require replication in other occupational groups.

In conclusion, our findings demonstrate the important role of manager support, assessed by manager psychosocial safety climate and manager behaviour, has in the mental health and well-being of ambulance personnel. Improvement of manager psychosocial safety climate and behaviour may be promising targets of team or organisational-level interventions that aim to improve the mental health of emergency services personnel.

## Supporting information

S1 File Questionnaire. (DOCX)

S1 Table Hierarchical regression analyses modelling the association between manager psychosocial safety climate, manager behaviour and mental health outcomes amongst ambulance personnel (valid full-time cases; n = 1172)*. *All values represent analyses with only full-time ambulance personnel employees selected (n = 1172 valid cases). Model 1: Unadjusted. Model 2: Adjusted for demographics (gender, age range, location, type of employment).
(DOCX)
