# Supplementary material for: The importance of manager support for the mental health and well-being of ambulance personnel
Source: PLoS One. 2018 May 23;13(5):e0197802. doi: 10.1371/journal.pone.0197802 (PMC5965892; doi:10.1371/journal.pone.0197802)
Supplement: S1 File — (DOCX) [file pone.0197802.s001.docx]

**S1 File. Questionnaire**

**Section 1:** (Wellbeing)

The Short Warwick-Edinburgh Mental Well-Being Scale (SWEMWBS) – 7 items

|  | **None of the time** | **Rarely** | **Some of the time** | **Often** | **All of the time** |
| --- | --- | --- | --- | --- | --- |
| I’ve been feeling optimistic about the future | 1 | 2 | 3 | 4 | 5 |
| I’ve been feeling useful | 1 | 2 | 3 | 4 | 5 |
| I’ve been feeling relaxed | 1 | 2 | 3 | 4 | 5 |
| I’ve been dealing with problems well | 1 | 2 | 3 | 4 | 5 |
| I’ve been thinking clearly | 1 | 2 | 3 | 4 | 5 |
| I’ve been feeling close to other people | 1 | 2 | 3 | 4 | 5 |
| I’ve been able to make up my own mind about things | 1 | 2 | 3 | 4 | 5 |

**Section 2:** (Psychological Distress)

Kessler Psychological Distress Scale (K6) – 6 items

The following questions ask about how you have been feeling during the **past 30 days**. For each question, please select the number that best describes how often you had this feeling.

| During the last 30 days, about how often did you feel… | **None of the time** | **A little of the time** | **Some of the time** | **Most of the time** | **All of the time** |
| --- | --- | --- | --- | --- | --- |
| 1. …nervous? | 1 | 2 | 3 | 4 | 5 |
| 1. …hopeless? | 1 | 2 | 3 | 4 | 5 |
| 1. …restless or fidgety? | 1 | 2 | 3 | 4 | 5 |
| 1. ...so depressed that nothing could cheer you up? | 1 | 2 | 3 | 4 | 5 |
| 1. …that everything was an effort? | 1 | 2 | 3 | 4 | 5 |
| 1. …worthless? | 1 | 2 | 3 | 4 | 5 |

**Section 3:** (Absenteeism/presenteeism)

World Health Organisation HPQ Absenteeism and Presenteeism questions (WHOHPQ)

1. How many days/shifts have you missed over the past 4 weeks (28 days) due to sickness absence
2. Over the last 6 months have you had a continuous 1 week period of sickness absence? Y/N
   1. If yes to 2: Was this due to mental health or emotional issues?
   2. Did your manager contact you during that 1 week period? Y/N
3. On a scale from 0 to 10 where 0 is the worst job performance anyone could have at your job and 10 is the performance of a top worker, how would you rate the usual performance of most workers in a job similar to yours?
4. Using the same 0-to-10 scale, how would you rate your usual job performance over the past year or two?
5. Using the same 0-to-10 scale, how would you rate your overall job performance on the days you worked during the past 4 weeks (28 days)?

**Section 4:** (Supervisor support at work)

These questions are about social support at work. Your answers are confidential and your workplace will not see any specific answers so please answer as honestly as possible. Please indicate how frequently each of these statements apply to you:

| My supervisor …. | **Never/ Hardly ever** | **Rarely** | **Sometimes** | **Often** | **Always** |
| --- | --- | --- | --- | --- | --- |
| … pays attention to my feelings and problems and notices if I’m not feeling so well | 1 | 2 | 3 | 4 | 5 |
| … shows that they appreciate the way I do my job | 1 | 2 | 3 | 4 | 5 |
| … supervisor helps me with a certain task if necessary | 1 | 2 | 3 | 4 | 5 |
| …. gives me advice on how to handle things if necessary | 1 | 2 | 3 | 4 | 5 |
| … would be someone I would speak to if I was experiencing workplace stress | 1 | 2 | 3 | 4 | 5 |
| … is considerate when managing team members | 1 | 2 | 3 | 4 | 5 |
| … involves me in decision making | 1 | 2 | 3 | 4 | 5 |
| … is accessible and approachable to people in the team | 1 | 2 | 3 | 4 | 5 |
| … remains objective when an issue between staff members arise | 1 | 2 | 3 | 4 | 5 |

**Section 5**: (Work Engagement)

Psychological Engagement Subscale from the NHS Work Engagement Staff Survey, based on the Utrecht Work Engagement Scale

The following statements are about how you feel while doing your work. Please read each statement carefully and decide how often you feel this way about your work. Indicate how often you experience this by placing a mark in the box that best describes how frequently you feel that way.

|  | **Never** | **Almost never**  (A few times a year or less) | **Rarely**  (Once a month or less) | **Sometimes**  (A few times a month) | **Often**  (Once a week) | **Very often**  (A few times a week) | **Always**  (Every day) |
| --- | --- | --- | --- | --- | --- | --- | --- |
| I look forward to going to work | 0 | 1 | 2 | 3 | 4 | 5 | 6 |
| I am enthusiastic about my job | 0 | 1 | 2 | 3 | 4 | 5 | 6 |
| Time passes quickly when I am working | 0 | 1 | 2 | 3 | 4 | 5 | 6 |

**Section 6**: Psychological Safety Climate - Management subscales from the PSC-12

The following statements concern the psychological health and safety in your workplace. Please answer with the best option provided.

|  | **Strongly disagree** | **Disagree** | **Neither agree or disagree** | **Agree** | **Strongly agree** |
| --- | --- | --- | --- | --- | --- |
| 1. In my workplace management acts quickly to correct problems/issues that affect employees’ psychological health |  |  |  |  |  |
| 1. Management acts decisively when a concern of an employees’ psychological status is raised |  |  |  |  |  |
| 1. Management show support for stress prevention through involvement and commitment |  |  |  |  |  |
| 1. Psychological well-being of staff is a priority for this organisation |  |  |  |  |  |
| 1. Management clearly considers the psychological health of employees to be of great importance |  |  |  |  |  |
| 1. Management considers employee psychological health to be as important as productivity |  |  |  |  |  |

**Section 7** (Demographics)

1. Age:

❑ <20

❑ 21-30

❑ 31-40

❑ 41-50

❑ 51-60

❑ 60+

1. Gender:

❑ Male

❑ Female

❑ Prefer not to say

1. Do you work for:

❑ ____________ (industry 1)

❑ ____________ (industry 2)

❑ ____________ (industry 3)

1. How long have you worked for _________[insert industry 1,2,3]:

❑ < 6 months

❑ 6 months – 1 year

❑ 1 to 5 years

❑ 5 to 10 years

❑ 10 to 15 years

❑ ≥ 15 years

1. Which location do you work at:

______________________(please specify)

1. Type of employment:

❑ Full time

❑ Part time

Thank you for your time. We will be in contact again by email in 6 months.

In the meantime, if you experience any psychological distress or would like to speak to someone about your own psychological health, please contact your EAP provider for support and to discuss. Alternatively you can call *beyondblue* Support Service Line 1300 22 4636 or Lifeline 13 11 14 which operate 24 hours a day 7 days a week, or see your GP who can assist with referrals to see a mental health professional.
