# Supplementary material for: The importance of manager support for the mental health and well-being of ambulance personnel
Source: PLoS One. 2018 May 23;13(5):e0197802. doi: 10.1371/journal.pone.0197802 (PMC5965892; doi:10.1371/journal.pone.0197802)
Supplement: S1 Table — *All values represent analyses with only full-time ambulance personnel employees selected (n = 1172 valid cases). Model 1: Unadjusted. Model 2: Adjusted for demographics (gender, age range, location, type of employment). (DOCX) [file pone.0197802.s002.docx]

S1 Table. Hierarchical regression analyses modelling the association between manager psychosocial safety climate, manager behaviour and mental health outcomes amongst ambulance personnel (valid full-time cases; n = 1172) *.

|  |  | | | | | |
| --- | --- | --- | --- | --- | --- | --- |
|  | **Manager Psychosocial Safety Climate (MPSC)** | | | **Manager Behaviour (MB)** | | |
|  | Beta (β) | r^2 /^ sr^2^ | p value | Beta (β) | r^2 /^ sr^2^ | p value |
| **Symptoms of CMD (K6)** | | | |  |  |  |
| Model 1: Unadjusted | -.331 | r^2^ = 0.13 | p<.01 | Unadjusted -.248 | r^2^ = 0.06 | p<.01 |
| Model 2: +Dem | -.386 | sr^2^ = 0.13 | p<.01 | +Dem -.260 | sr^2^ = 0.06 | p<.01 |
| **Well-being (SWEMWBS)** | | | |  | | |
| Model 1: Unadjusted | .350 | r^2^ = 0.12 | p<.01 | Unadjusted .316 | r^2^ = 0.10 | p<.01 |
| Model 2: +Dem | .397 | sr^2^ = 0.13 | p<.01 | +Dem .322 | sr^2^ = 0.09 | p<.01 |

***** All values represent analyses with only **full-time** ambulance personnel employees selected (n = 1172 valid cases).

**Model 1:** Unadjusted.

**Model 2:** Adjusted for demographics (gender, age range, location, type of employment).
